# Genioplasty for genioglossus muscle advancement in patients with obstructive sleep apnea-hypopnea syndrome and mandibular retrognathia

**DOI:** 10.1016/S1808-8694(15)30099-9

**Published:** 2015-10-19

**Authors:** João Ferreira dos Santos Junior, Márcio Abrahão, Luiz Carlos Gregório, Adriane Iurk Zonato, Emne Hammoud Gumieiro

**Affiliations:** a M.S. in Health Sciences - UNIFESP, Adjunct Professor - School of Dentistry - Universidade de Santo Amaro UNISA.; b Associate Professor of Otorhinolaryngology-Head and Neck Surgery - UNIFESP.; c PhD in Otorhinolaryngology and Head and Neck Surgery - UNIFESP, Professor of Otorhinolaryngology - UNIFESP.; d PhD in Otorhinolaryngology and Head and Neck Surgery - UNIFESP, Co-Advisor of Otorhinolaryngology and Head and Neck Surgery - UNIFESP.; e M.S. in Orthodontics - UNICASTELO, PhD student in Health Sciences - Otorhinolaryngology-Head and Neck Surgery - UNIFESP.; Disciplina de Otorrinolaringologia e Cirurgia de Cabeça e Pescoço da UNIFESP - Universidade Federal São Paulo - São Paulo SP, Brasil.

**Keywords:** genioplasty, mandibular retrognathia, sleep apnea syndrome, surgical treatment

## Abstract

Obstructive sleep apnea syndrome is a complex disease, which the etiology is multifactorial and incompletely understood. Surgery with genioglossus advancement is indicated in hypopharynx obstruction.

**Aim:**

evaluate the efficacy and complications of genioplasty technique for genioglossus muscle advancement in patients with obstructive sleep apnea-hypopnea syndrome (OSAHS).

**Methods:**

polysomnography, physical examination and cephalometric analysis were performed in 10 non-obese patients, who presented mild or moderate OSAHS, an apnea-hypopnea index (AHI) between 5 and 30, with hypopharynx obstruction and mandibular retrognathia.

**Results:**

the AHI preoperative mean of 12.4±4.6 decreased to a postoperative mean of 4.4±5.7 (p < 0,001). When 50% reduction of preoperative AHI was chosen as a parameter, its rate was 70% (7/10). Cephalometric analysis revealed an increase in the posterior airway space (PAS) in all patients, from a preoperative mean value of 7.9±2.3mm to a postoperative mean value of 10.8±2.5mm (p < 0,001).

**Conclusion:**

genioplasty for genioglossus advancement seemed to reduce OSAHS signs, thus, it can be considered as an option for the surgical treatment of patients with hypopharynx obstruction. Data collected supports this surgical procedure as an option in patients with OSAHS and mandibular retrognathia.

## INTRODUCTION

Sleep Obstructive Apnea Syndrome (SOAS) is considered the most common sleep obstructive disorder, and it affects millions of people. It is associated with significant morbi-mortality, causing physiologic alterations in the cardiovascular and respiratory systems as a result of hypoxia and repeated awakenings during sleep^1^. Sleep fragmentation causes excessive daily sleepiness, fatigue, limited attention span, less daily energy - which may cause work and automobile accidents^2^.

Dento-facial deformities are considered an important risk factor in non-obese SOAS patients and include mandibular and maxillary retrognatism, retroposition of the tongue and increase in anterior third of the face. Mandibular retrognatism may be one of the factors responsible for the reduction in posterior airway space (PAS) and reduction in the distance between the hyoid bone and the mandibular plane, causing hypopharynx obstruction^3^, ^4^.

Surgical reconstruction of the upper airway may be carried out in one or more pharyngeal regions, especially in cases of hypopharynx and tongue base hypopharynx, a surgical technique described by Riley et al.^5^. This procedure is based on mandibular osteotomy to bring the genioglossal muscle forward, causing tongue pulling, avoiding upper airway obstruction in the hypopharynx during sleep.

In this study, the efficacy and complications associated with the mentoplasty approach to bring forward the genioglossal muscle will be assessed in patients with SOAS and mandibular retrognatism.

## MATERIALS AND METHODS

This study was analyzed and approved by the Institution’s Ethics and Research Committee, under protocol # 445/01 and carried out in 10 patients of both genders with ages above 18 years, diagnosed with SOAS by polysomnography, with clinical and cephalometric diagnosis of mandibular retrognatism. The patients were referred from the Sleep Institute of the Psychobiology Department to the Department of Otorhinolaryngology in charge of the surgical treatment.

Patient inclusion criteria were the following:

1. Hypopnea and apnea index above 5 and below 30
2. Patients with BMI below 30 Kg/m2
3. Patients with hypopharynx obstruction (Type III) or oropharynx and hypopharynx (type II) obstruction according to the Fujita et al.^7^
4. Mandibular retrognatism
5. Patients complaining of nasal obstruction or with nasal obstruction that may be corrected during surgery.

Patient exclusion criteria were as follows:

1. Patients with obstruction in the oropharynx only (Type I) according to the classification by Fujita et al.^7^
2. Patients with normal facial profile or patients with mandibular prognathism
3. Chronic obstructive pulmonary disease
4. Neurological or psychiatric disorders
5. Sleep apnea of central origin
6. Use of sleep induction medication

### Preoperative Assessment

Night time polysomnography includes the following parameters: electroencephalogram, chin and tibia electromyography, thorax and abdomen movements, oral and nasal airflow, oxyhemoglobin level, electrooculogram and electrocardiogram. Patients with mild and moderate SOAS, hypopnea-apnea index (HAI) varying between 5 and 30, were included in this study.

Otorhinolaryngological evaluation was based on nasal cavity rhinoscopy and nasofibroscopy, together with cephalometric analysis, as described by Riley et al.^6^. Hypopharynx obstruction happened when the tongue base hit the posterior pharyngeal wall and impaired larynx visualization or if the cephalometric analysis showed any PAS narrowing (PAS smaller than 10 mm).

Mandibular retrognatism was carried out by Macnamara’s cephalometric analysis^8^ and facial assessment of soft tissue by Arnett e Bergman’s9 analysis. During facial analysis, we used the facial profile angle (FPA), which is formed by the lines that pass through the soft tissue points: glabella, subnasal and pogonion. When the FPA is less than 165º characterizing mandibular retrognatism.

**Figure 1:**
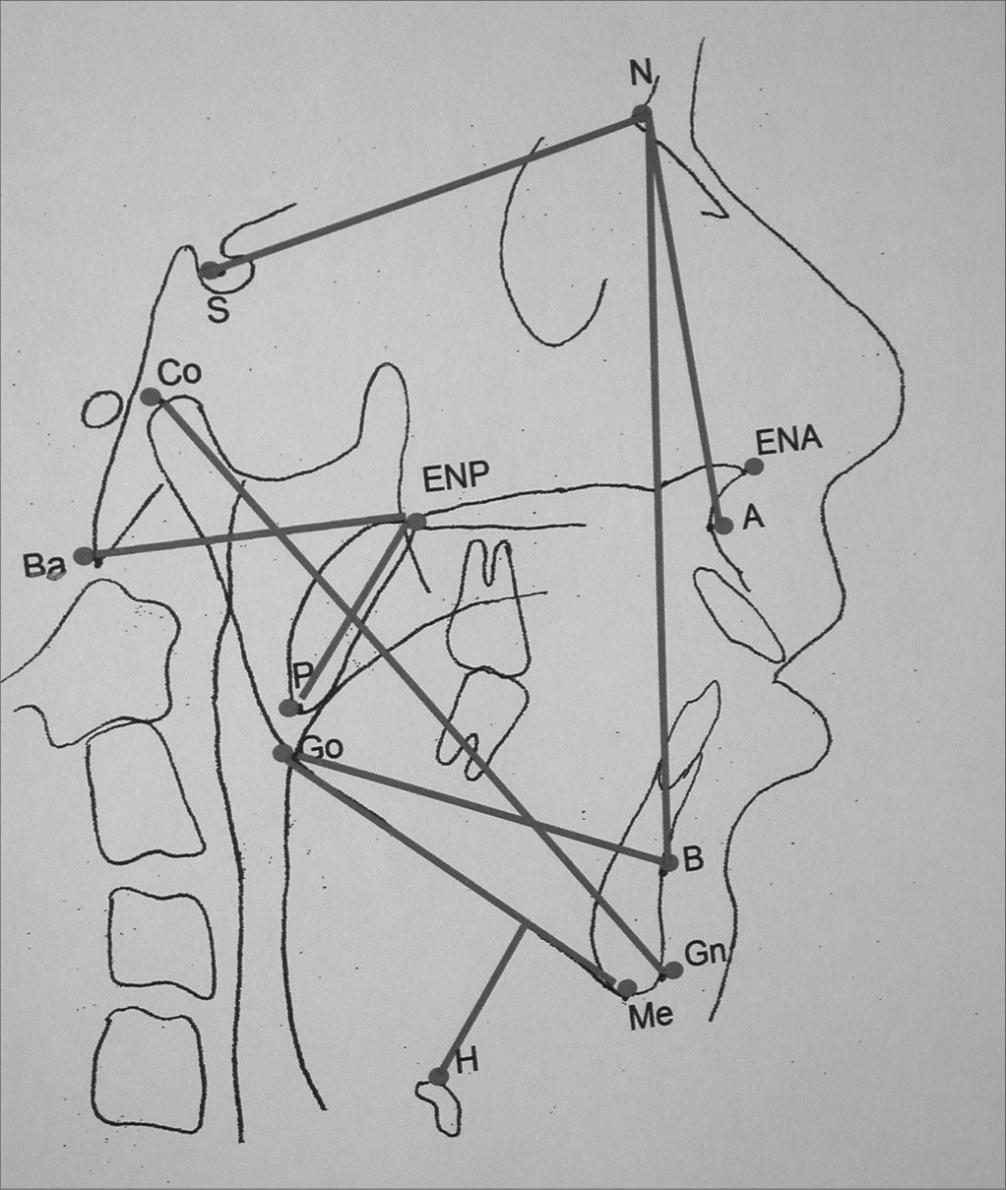
Cephalometric measures of dentofacial morphology and pharyngeal air space. Posterior Air Space (PAS)- Distance between the tongue base and the posterior pharyngeal wall, determined by the B-Go line. Distance Hyoid-mandibular plane - distance from the H point - anterior and superior most point of the hyoid bone all the way to the mandibular plane (Me-Go), through a line perpendicular to it. Soft Palate Length - Distance between the ENP-P Ba-ENP points-Bony pharynx size. Distance between the Ba-ENP points Mandibular length (Co-Gn)- Distance between the condylar point and the gnathion in mm. SNA- Antero-posterior maxilla position in relation to the skull base. Angle formed by the S-N and N-A lines. SNB- antero-posterior mandible position in relation to the skull base. Angle formed by the S-N and N-B lines.

Cephalometric analysis of dentofacial morphology and pharyngeal air space may be seen in Figure 1.

### Surgical Technique

In order to perform the mentoplasty, we make an incision on the bottom of the gengivolabial groove of the canine tooth on the right side all the way to the canine tooth on the left side. Mucoperiosteal detachment is carried out in the chin region all the way to the mandible body, avoiding to damage the mentonian nerve. The muscle insertion on the mandible internal cortical bone is preserved in order to assure the vascularization of the osteotomized segment and thus keep effective traction of the genioglossal muscle.

**Figure 2:**
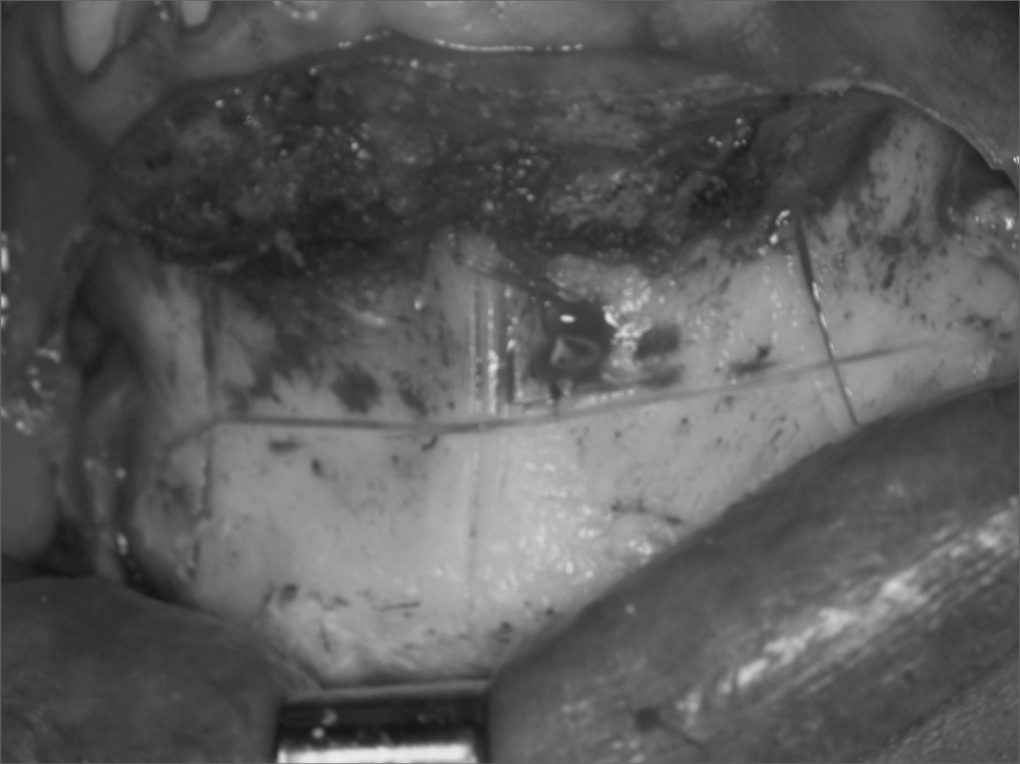
Mentoplasty. Osteotomy for genioglossal muscle advance

**Figure 3:**
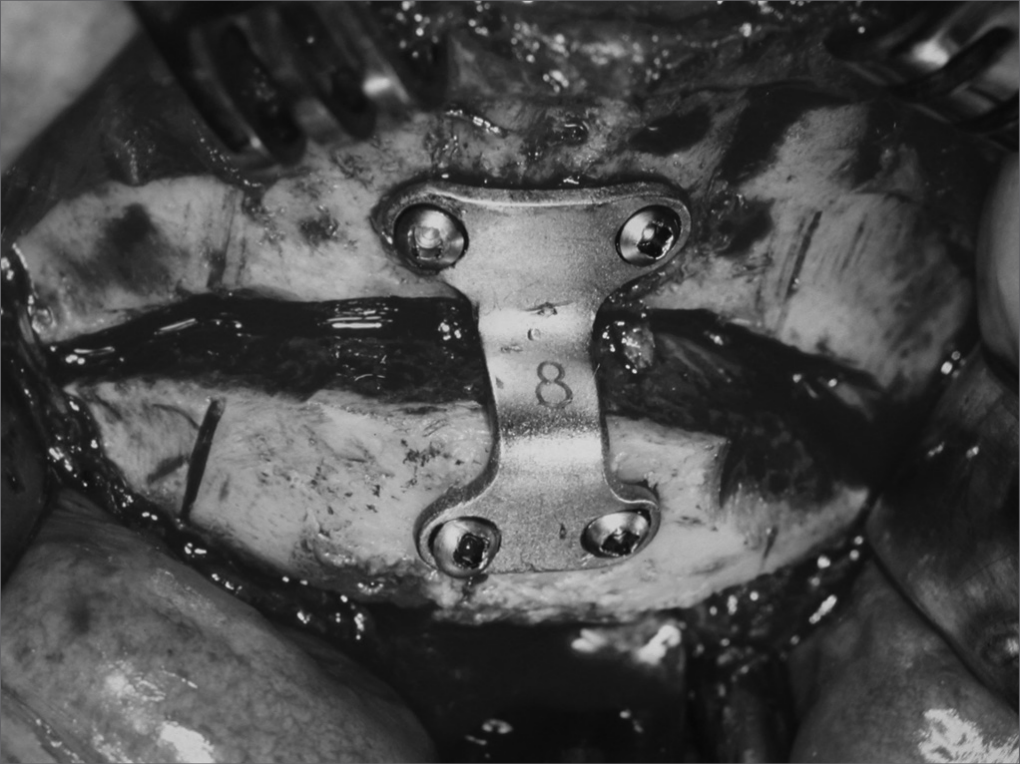
Mentoplasty. Osteosynthesis with rigid internal fixation

After proper exposure of the mandibular symphysis, we did three vertical lines, one medial and two laterals, for reference purposes during chin advance, avoiding asymmetries during osteosynthesis. We carried out a horizontal osteotomy below dental apexes, and the upper one was between the right and left canine teeth; osteotomy extends posteriorly, tilting downward, passing five millimeters below the chin foramen, and thus avoid damaging the mentonian nerve, going all the way to the first molar region (Figure 2). After completing the bicortical osteotomy, we mobilized the distal segment of the antero-inferior segment. At this point, we should not cut the genioglossal, geniohyoid and digastric muscles, aiming at increasing posterior airspace, since we may pull these muscles with anterior chin repositioning. After anterior mobilization of the distal segment we fix this segment in the horizontal direction with the largest possible advance, allowing the internal bone cortical to touch the external cortical of the proximal segment, thus allowing bone consolidation and an effective advance of the genioglossal muscle. Osteotomy fixation can be carried out by means of an internal rigid fixation with plate and screws that allow bone stability and consolidation for the distal segment. The osteosynthesis material used was the Paulus plate in a “double L” shape with two holes in each one of the horizontal segments, with four 2.0mm diameter screws (Figure 3).

All patients received 10mg endovenous infusion of dexamethasone and 1g cephalexin after anesthesia induction.

**Figure 4:**
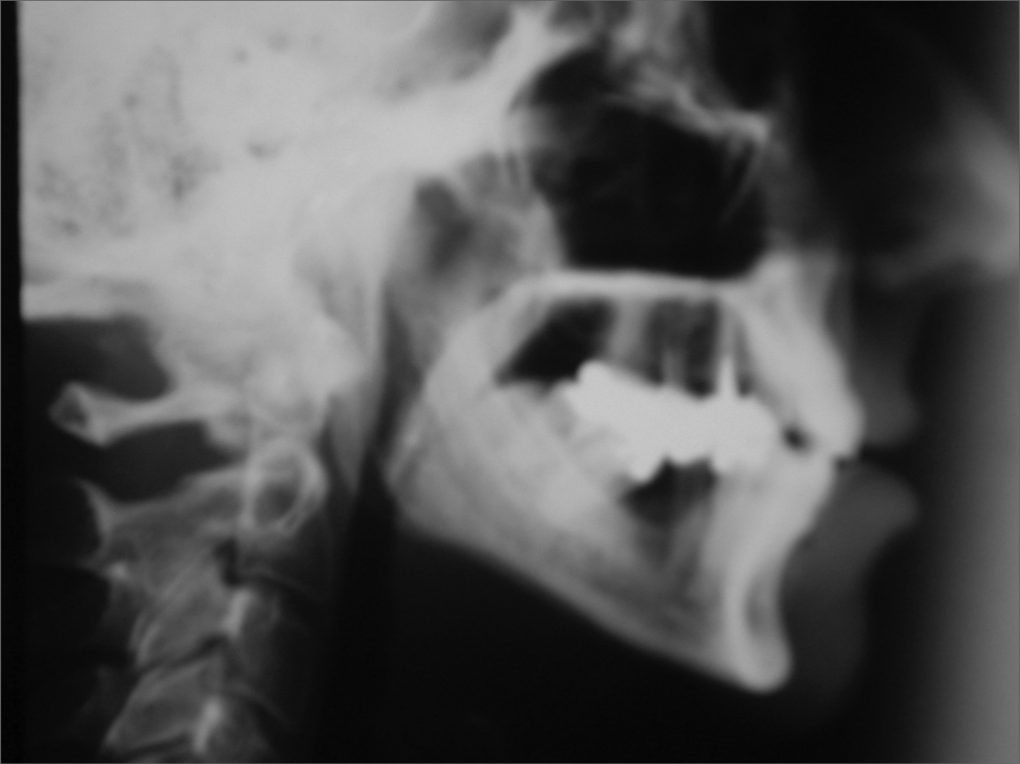
Teleradiography. Preoperative

**Figure 5:**
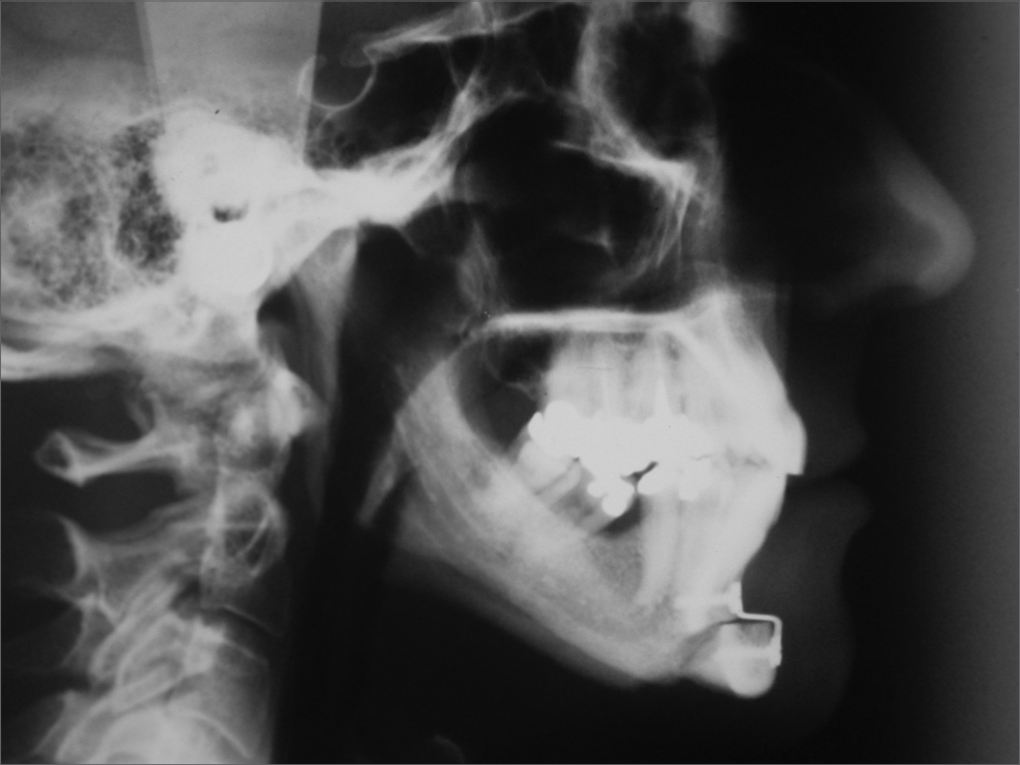
Teleradiography. Postoperative

Preoperative and postoperative side view radiographs were taken for control purposes (Figures 4 and 5).

Patients with oropharynx obstruction (Type II) underwent uvulopalatopharyngoplasty. Patients that had some anatomical alteration in the nasal cavity underwent concurrent surgical correction, as shown on Table 1.

**Table 1:** Preoperative assessment and procedures carried out.

Patients	Age (years)	Gender	APF (degrees)	Obstruction Type	Complementary Surgery	Advance (mm)
1	30	male	151	III	Septoplasty	10
2	46	male	152	II	UPFP	10
3	46	male	163	II	UPFP	10
4	57	male	161	III	Previous UPFP	8
5	50	male	163	II	UPFP	8
6	31	male	154	II	UPFP	10
7	52	male	163	II	UPFP	8
8	54	fem.	156	III	Previous UPFP	10
9	40	fem.	154	II	UPFP	8
10	54	fem.	155	III	Septoplasty	8

APF: Facial profile angle (in degrees); UPFP: uvulopalatopharyngoplasty

### Postoperative Assessment

Postop assessment was carried out on the seventh and thirtieth days, in order to assess complications and tissue repair. Polysomnographyc (Table 2) and cephalometric analyses (Table 3) were carried out between the fourth and the sixth months of postoperative.

**Table 2:** Pre and postoperative assessment of polysomnographic parameters (n=10).

Patients	Pre and Post BMI	Pre and Post HAI	Pre and Post SaO2min	Pre and Post REM
1	27,3 28,5	21 1	86 91	30,0 20,6
2	27,4 30,3	14 1	85 93	13,4 21,9
3	29,1 28,0	9,8 3	77 87	21,7 31,6
4	28,0 29,4	10 1	89 85	8,4 0,0
5	25,0 26,0	7,4 6	86 92	17,1 28,0
6	30,0 29,4	16 4	59 86	20,8 24,3
7	23,5 24,6	8 6	85 85	13,4 19,0
8	22,0 23,0	16 7	85 93	8,8 19,9
9	23,0 22,0	7,3 3	89 90	22,6 21,4
10	22,0 22,0	13,7 12	87 90	0,0 16,0
Mean	25.36 26.32	12,38 4,40	82.88 88.4	15.63 20.29
SD	±2.98 ±3.23	±4.62 ±5.7	±9.04 ±3.13	±8.66 ±8.43

BMI: Body Mass Index (kg/m); HAI: Hypopnea and Apnea Index (for each hour of sleep); min SaO2: minimum oxygen saturation; REM: sleep stage -rapid eye movement; Pre: preoperative; Post: postoperative.

**Table 3:** Preoperative assessment of cephalometric parameters (n=10).

Patients	SNA	SNB	Co-Gn Pre Post	H-PM Pre Post	PAS Pre Post
1	81	79	127 135	32 30	11 13
2	86	78	112 122	38 34	12 14
3	78	74	120 126	34 32	7 11
4	92	84	132 140	24 24	6 9
5	77	72	111 120	26 26	10 12
6	89	83	119 122	20 15	8 15
7	82	72	120 125	19 18	8 9
8	83	76	112 120	18 18	5 8
9	76	67	112 118	22 16	6 9
10	77	72	106 111	22 20	6 8
Mean	82.1	75.7	117.10 123.9	25.5 23.3	7.9 10.8
DP	± 5.47	±5.36	±8.02 ±8.35	±6.88 ±6.93	±2.38 ±2.57

SNA: antero-posterior maxilla position (in degrees); SNB: antero-posterior mandible position (in degrees); Co-Gn: mandibular length (in millimeter); PAS: Posterior air space; H-PM: distance between the mandibular plane and the hyoid bone (in millimeters). Pre: preoperative; Post: postoperative.

Treatment success criterion was established according to the following parameters:

1- 50% reduction in preop HAI.
2- Postop HAI below five.
3- Minimum postoperative oxygen saturation (SaO2) above 90%.

Statistical analysis was carried out by means of the t-paired test in order to check for differences between variables in the pre and postoperative.

## RESULTS

Postoperative polysomnography was carried out in a period that varied between 4 and 6 months. Average pre and postop HAI were 12.4 ±4.6 e 4.4 ±5.7, respectively, and the difference was statistically significant (p < 0.001). All patients had reductions in their initial HAI. Success rate was of 60% when HAI was below five; and 70% success rate when the reduction criterion was of 50% in the preoperative index.

Pre and postoperative SaO^2^ mean values were of 82.8 ±9.0 and 88.4 ±3.1 respectively, showing a statistically significant difference (p < 0.05). Postoperative SaO^2^ above 90% was found in 60% of the patients.

Pre and postoperative REM sleep average duration was of 15.6 ±8.6 and 20.2 ±8.4 respectively, and such differences were not statistically significant. Also, there was no statistically significant difference in other parameters of the polysomnography exam such as: sleep efficacy, number of micro-awakenings, upper and lower limb movements and sleep stages.

Facial side view x-ray for cephalometric analysis was also characterized in the aforementioned period. Pre and postoperative PAS mean distances were 7.9 ±2.3mm and 10.8 ±2.5mm, respectively, with a statistically significant increase (p < 0.001). In all patients there was PAS increase.

H-PM mean distance was 25.5 ±6.8mm and 23.5 ±6.9mm, resulting in statistically significant reduction (p < 0.01). In maxillo-mandibular ratio we found the following preoperative values: mean SNB of 75.7 ±5.3°, mean SNA of 82.1 ±5.47° and mean ANB of 6.4 ±2.1°. Mean preoperative mandibular length (Co-Gn) was of 117.1mm and mean postoperative of 123.9 ±8.3mm, showing a statistically significant increase (p < 0.001).

Patients underwent mentoplasty alone or associated with nasal surgery were hospitalized for two days, and patients who underwent mentoplasty associated with uvulopalatopharyngoplasty stayed, in average, three days in the hospital.

Upper lip paresthesia, edema, ecchymosis and suture dehiscence were the temporary postoperative complications observed, only one patient had lower lip paresthesia for less than 4 months, because of a local infection which was treated with antibiotic therapy and wound care. Postoperative healing happened between 3 and 6 weeks.

## DISCUSSION

Dentofacial deformity is one of the most important risk factors in non-obese SOAS patients, causing airway obstruction and the possibility of surgical treatment. The most common characteristics are mandibular and maxillary retrognatism, tongue posteriorly positioned and facial length increase^3^, ^4^. Mandibular retrognatism can be one of these factors and may reduce the posterior air space in the hypopharynx region4.

Genioglossal muscle advance is a less invasive surgical approach, which can be indicated in patients with light and moderate SOAS^5^, ^11^, ^15^. Moreover, genioglossal muscle advance in patients with hypopharynx obstruction can be the first choice in surgery, because of its low morbidity.

The surgical treatment presented in this study caused the advance of the genioglossal, geniohyoid and digastric muscles. Its advantage is the possibility of visualizing the inner bone cortical, where the muscles are inserted, and avoiding muscle rotation during its advance. The only disadvantage is the possibility for mandible horizontal fracture^13^; however, by using very thin saws for osteotomy and stabilizing the advanced segment with rigid internal fixation reduces this problem^16^. In the present investigation, rigid internal fixation caused proper bone growth between the osteotomized segments. The usual genioglossal muscle advance osteotomy is based on a quadrangular bicortical osteotomy of the anterior mandible region involving the genial tubercle and genioglossal muscle advancement, without causing dental occlusion alteration and maxillo-mandibular advance11. However, since this technique bears some disadvantages, one of them is that during the advance procedure it is necessary to rotate the muscles in 90º and this could seriously impair its function. Another complication of the technique is to injure the genioglossal muscle insertion during osteotomy. The mentoplasty technique described here allows one to see the inner mandible cortical bone and it also keeps the advanced segment vasculature.

The posterior air space (PAS) is an important parameter used to determine air space reduction in the hypopharynx region. A PAS below 7mm indicates hypopharynx obstruction^14^ and when it comes associated with an H_PM distance greater than 20mm, the patient has severe mandibular retrognatism^3^, ^6^, ^10^, ^13^, ^14^. Our results showed that a pre and postoperative PAS of 7.9±2.3mm and 10.8±2.5mm, respectively, indicate a statistically significant increase (p < 0.001). This increase seen in all patients is also reported in the literature^3^, ^6^, ^11^, ^12^.

The mentoplasty technique used to advance the genioglossal muscle in patients with mandibular retrognatism caused PAS increase and an advance of the soft tissue present in the mentonian region, and may even be observed in the Co-Gn distance, which corresponds to the mandibular length^16^, ^20^. Moreover, the change in osteotomy with minimum morbidity may result in a more cosmetically pleasing facial profile, with more patient satisfaction for those young and non-obese subjects, which respond better to facial profile changes. Nonetheless, in normofacial patients or in those with mandibular prognathism we indicate the approach described by Riley et al.^5^.

Many treatment success parameters are described in the literature;^7^, ^12^, ^14^, ^17^ however the majority of those used are HAI and minimum SaO^2^ values, especially pre and postoperative values comparison. Riley et al.^17^ carried out an advance in the genioglossal muscle and myotomy elevating the hyoid bone in 55 patients, and considered a successful treatment those patients that had a minimum SaO2 > 90%, HAI < 20 or a 50% reduction in initial HAI: success rate based on these issues was of 67%. Ramirez & Loube^14^ carried out genioglossal advance, hyoid bone elevation and uvulopalatopharyngoplasty in non-obese patients and, by using the same parameters had a success rate of 42%. Johnson & Chinn^12^ considered the treatment to be of clinical success when the postoperative HAI is below 5 and 10, or with a 50% reduction in initial HAI/they had a success rate of 66% and 78%, respectively, in a HAI < 5 and HAI < 10; and 78% of success rate when 50% reduction in initial HAI was the criterion chosen.

In our investigation, success criteria included a HAI below 5, 50% reduction in initial HAI and minimum SaO^2^ > 90%. Our results indicated that 60% of the patients had HAI < 5; 70% with 50% of initial HAI reduction; and 60% with minimum SaO^2^ > 90%. All patients had postoperative HAI reduction. Despite the results, there is great difficulty in determining success rates in SOAS patients if only HAI is chosen as a success criterion. Sleep stages, excessive daily sleepiness and other cognitive parameters may be considered as indicators of surgical treatment morbidity and success.

Postoperative complications stemming from the mentoplasty technique are similar to those found in the chin rectangular osteotomy for genioglossal muscle advance, as we can see in the literature^17^, ^19^, ^20^.

Possible failure in treatment approach is brought about by severe retrognathism or obstructions in other airway regions, which were not diagnosed in preoperative evaluation. Patients who do not respond favorably to the proposed treatment had SNB angle of 72º. According to our results and reports in the literature, patients with severe mandibular retrognatism may fail genioglossal muscle advance and uvulopalatopharyngoplasty surgical treatment; in such cases it is worth considering maxillo-mandibular advance^18^.

SOAS is multifactorial and not fully understood, and does not have an ideal surgical treatment. The surgical techniques described in the literature for SOAS must consider it in a case-by-case basis, observing body mass index, apnea severity, region obstructed, dentofacial deformity severity. Surgical treatment must be tailor-made and SOAS remains a challenge for health care professionals.

## CONCLUSION

The surgical approach to advance the genioglossal muscle is useful in the treatment of non-obese patients with SOAS and hypopharynx obstruction.

This technique maintains the osteotomized segment vasculature, without muscle rotation, allowing for effective genioglossal muscle traction. The limitation of such procedure is accurate indication, in which the patients must have mandibular retrognathism.
